# The association between resilience resources, contextual factors and mental health status: a national population-based study

**DOI:** 10.1186/s12889-022-13013-2

**Published:** 2022-03-29

**Authors:** Foteini Tseliou, Pauline Ashfield-Watt

**Affiliations:** 1 Wales Institute of Social and Economic Research and Data, Cardiff University, 38 Park Place, Cathays, Cardiff, Wales CF10 3BB UK; 2 HealthWise Wales, Neuadd Meirionnydd, University Hospital of Wales, Cardiff University, Heath Park, Cardiff, Wales CF14 4YS UK

**Keywords:** Coping, Resilience, Cohesion, Neighbourhood, Mental health, Carers

## Abstract

**Background:**

Although a range of risk factors have been linked with poor mental health across the population, the underlying pathways leading to mental ill health remain unclear. There is a need to investigate the effects and interplay of both protective and risk factors. This population-based study aimed to explore the effects of individual and contextual factors on mental health status. Record-linkage was implemented between health and lifestyle data drawn from HealthWise Wales (HWW), a national population health survey of people > 16 years who live or access their healthcare in Wales, and treatment data from primary healthcare records. Mental health status was assessed using three different measures, including the self-reported MHI-5 and WEMWBS scales and mental health treatment in electronic healthcare records (EHR).

**Result:**

Using cross-sectional data from 27,869 HWW participants aged over 16 years, lifestyle factors, resilience, social cohesion and neighbourhood attraction were associated with mental health across all measures. However, compared to contextual factors, the cluster of individual factors was more closely associated with poor mental health, explaining more of the variance across all measures used (MHI-5: 9.8% versus 5.4%; WEMWBS: 15.9% versus 10.3%; EHR: 5.5% versus 3.0%). Additional analysis on resilience sub-constructs indicated that personal skills were the most closely correlated with poorer mental health.

**Conclusion:**

Mental health status was more closely linked with individual factors across the population than contextual factors. Interventions focusing on improving individual resilience and coping skills could improve mental health outcomes and reduce the negative effect of contextual factors such as negative neighbourhood perceptions.

**Supplementary Information:**

The online version contains supplementary material available at 10.1186/s12889-022-13013-2.

## Background

Mental health status is shaped through the cumulative effect of risk and protective factors which can affect the individual’s ability to adapt to new situations and develop healthy coping mechanisms. A complex interplay of individual and societal factors can lead to variation in mental health status across the population. Common mental disorders are expected to become the most common diseases by 2030 [1], with recent studies suggesting that one in five people in the world will experience a mental disorder in their lifetime [2]. This highlights the need to focus research on better understanding risk and protective factors of mental health outcomes and ways of supporting those who are at risk of experiencing poor mental health.

Resilience has been previously described as the ability to bounce back from negative life events and function normally, using self-regulation and cognitive coping skills when faced with stressful situations to reduce the deleterious effects on the individual and maintain their wellbeing [3]. Recent definitions have expanded the scope of resilience as a socioecological construct separating it from psychological resilience (or ego-resilience) and highlighting the interplay between individual processes and contextual factors [4]. The need for capacity to negotiate intra- and interpersonal resources to achieve “high” resilience in the face of adversity is further reflected by the re-conceptualisation of resilience as a construct that is shaped by individual attributes, personal relationships and a sense of belonging [5].

Resilience has been linked with demographic characteristics such as increasing age and female gender, with women also being more at risk of poor mental health outcomes [6]. The “cost of caring” hypothesis suggests that women are at an increased risk of mental ill health due to experiencing chronic stress linked to adverse life events [7]. Although individual factors such as resilience may be closely associated with mental health, interpersonal and contextual factors such as the neighbourhood environment might moderate the link between individual characteristics and mental wellbeing [8]. Socio-economic status, both at household and community levels, has been associated with perceived social capital and cohesion, which can significantly affect physical and mental health [9, 10]. Additionally, low levels of social cohesion have been linked to unhealthy behaviours such as alcohol use [11] and poor self-rated health [12]. Providing informal care for a family member or friend, despite having been previously used as an index of resilience [13], can also be a contributing factor to poorer mental health, especially for younger individuals [14].

Across different settings and samples, intrinsic characteristics such as cognitive coping styles have been shown to moderate the potential effect of adverse experiences acting as protective factors to mental wellbeing [15, 16], while physical health and engagement to physical activities or exercising have been psychological resilience thus better physical and mental health especially among older adults [17, 18]. Beyond the individual and household-level factors, a range of wider social predictors including official records and resident perceptions on the characteristics of the living environment including neighbourhood crime and violence have been linked with poor mental health both through direct and indirect pathways such as victimisation or development of maladaptive coping strategies [19–21]. This association is more pronounced within areas with low levels of social cohesion [22] where perceived lack of safety might lead to loneliness and reduced wellbeing [23]. However, a number of previous studies have examined risk factors in isolation and within small selective sub-populations (for example with partners/spouses or older people), limiting their generalisability to other settings [24, 25] and causing issues in the implementation of complex models [26]. There are also questions regarding the epidemiological validity of existing evidence as this largely depends on studies using self-reported assessment of mental health in the form of survey data [9, 10], postal questionnaires for cohort samples [25] or Census returns for population-based samples [27]. This limitation could be overcome by including different types of assessment tools, such as measures based on diagnostic criteria and healthcare records, which are not limited by report bias and could potentially help explain the current lack of consensus on population mental health assessment.

Though the interrelation of individual and contextual factors has been explored previously, knowledge of the specific psychosocial pathways leading to poor mental health remains limited. There is a need to understand both the independent effects of individual and contextual factors, as well as their cumulative effect on population mental health. Therefore, the use of population-based data which provide a wide range of socio-demographic characteristics and other risk factors closely linked to mental health status could provide deeper understanding of the underlying mechanisms which may lead to mental ill health.

### Aims

The aim of the study was to investigate associations between individual and contextual factors and the experience of poor mental health in a national population survey.

Our objectives were therefore to: i) describe the prevalence of mental ill health within the cohort using three validated measures and characterise individual and contextual factors purported to affect mental health status ii) explore associations between individual (including resilience resources) and contextual factors (including social cohesion and neighbourhood attraction), and poor mental health status, iii) investigate how these factors might be differently associated with perceived mental health and mental health treatment within our cohort iv) investigate the influence and interplay of these factors in relation to mental health in a sub-group (informal carers) exposed to chronic stress.

## Methods

### Data sources

The guidelines of the Strengthening the Reporting of Observational Studies in Epidemiology (STROBE) (www.strobe-statement.org/fileadmin/Strobe/uploads/checklists/STROBE_checklist_v4_cross-sectional.pdf) checklist of items were accounted for by the research team during the implementation of the present study. The primary data source for this study were baseline data from HealthWise Wales (HWW), Wales’ national longitudinal health study which is funded by Health and Care Research Wales. The Wales Research Ethics Committee (REC) 3 approved the HWW study on 16/3/2015 (reference 15/WA/0076; quinquennial review 20/WA/0064). The study was launched in May 2015 and comprises adults (> 16 years) who live in Wales or access their healthcare in Wales [28]. All HWW participants, provided informed consent for their data to be collected via questionnaire modules hosted on the HWW platform and to be linked anonymously to their healthcare records. These data were linked via Secure Anonymised Information Linkage (SAIL) to routinely collected healthcare records and stored on the Secure Analysis Portal and Protected HWW Information Repository (SAPPHIRe), through which all data are accessed by approved researchers on a project basis [28]. At the time of analysis, 28,983 participants had provided information on key variables that facilitated confident linkage to their healthcare records using gender, date of birth and geographical identifiers (893 individuals dropped due to fuzzy matching to health records; match rate: 97.01%).

### Cohort characteristics

This study presents the information on self-reported mental health from the HWW questionnaires and evidence for common mental health disorders (CMD) identified from primary healthcare records. Personal characteristics drawn from HWW data included gender and age, with individuals grouped at 10-year age groups. Wales is ethnically relatively homogenous, therefore, two ethnic groups (white, non-white) were defined.

### Caring status

The level of caring responsibilities and the impact of caring on employment were assessed through self-report questions: i) “*Do you look after, or give any help or support to family members, friends, neighbours or others because of long-term disability, mental health disability, problems related to old age?”*, with participants grouped as providing 0; 1–19; 20–49 and 50 + hours of care per week, and ii) *“Have you ever had to give up work to look after a family member, friend, or neighbour?”* (Yes/No).

### Individual factors

Health behaviours were investigated as individual lifestyle behaviours which could affect mental health and wellbeing. These behaviours included: drinking above guidelines (Yes/No), defined by the Welsh Health Survey as > 3 daily units for a man and > 2 daily units for a woman. Physical inactivity was estimated using the self-report General Practice Physical Activity Questionnaire (GPPAQ) [29], with participants being grouped as physically inactive and active. Smoking status, including cigarettes, hand-rolled, pipe or cigars (grouped as smoker & non-smoker) and eating habits (grouped as unhealthy and healthy eating) were assessed using self-report questionnaires.

The Resilience Research Centre Adult Resilience Measure (RRC-ARM) assesses the different levels of resources (including individual, interpersonal, community and cultural resilience) [5] which individuals can use to cope with significant adverse experiences. In the present study, a 28-question variant for adults with three response levels was selected for self-completion online. In this study, RRC-ARM 28 was used as a continuous scale, with higher scores signifying higher levels of resilience-associated characteristics (also see distribution across the study population in Supplementary Fig. 1). In accordance with the RRC-ARM 28 scale user manual [30], to allow for a more precise investigation of different aspects of resilience resources, we constructed three subscales: individual; personal relationships with key individuals; and context or sense of belonging.

### Contextual factors

Relationship status was considered as a contextual factor (in relationship, single) as it is thought of as a form of social support, with married/ cohabiting individuals previously shown to have better social networks and less likely to report poor mental health [31].

Socio-economic status was determined using occupational social class/ employment derived using the National Statistics Socio-Economic Classification (NS-SEC) [32] and grouped as higher, intermediate and lower-level positions, with a separate category for students and long-term unemployed. Two area indicators were included: a classification of population density based on the lower layer super output areas in Wales [33] and area-level deprivation which was based on the Welsh Index of Multiple Deprivation [34] and calculated on the quintile level from most to least deprived area (quintile 1 = most deprived).

Buckner’s neighbourhood cohesion scale [35] with 17 items was also completed by HWW participants. Question 8 “I think I agree with most people in my neighbourhood about what is important in life” and 16 “A feeling of fellowship runs deep between me and other people in this neighbourhood” were not included, in accordance with previous studies conducted in similar data [10, 36], though question 18 “Overall I think this is a good place to bring up children” was included. As the scale has been reported to be unidimensional [36], factor analysis with principal components analysis followed by a varimax rotation (Spearman’s rank) were used to identify a set of underlying common factors (see full analysis steps in Supplemental Fig. 2 and Supplemental Table 1). The two identified components (neighbourhood attraction and social cohesion) were used to assess the level of neighbourhood cohesion across the cohort.

### Mental health measures

Mental health was assessed using three measures which explored self-report mental health and wellbeing as well as mental health treatment.

The five-question Mental Health Inventory (MHI-5) score is a brief questionnaire that has been used to screen for depressive symptoms in various settings [37]. It consists of five questions focusing on depressive and anxiety symptoms experienced during the four weeks prior to questionnaire completion, with six possible responses ranging from “all of the time” (1 point) to “none of the time” (6 points). The MHI-5 score is calculated by summing the scores and scaling the results to a 100-point scale which is subsequently dichotomised (case/non-case) using a cut-point of 60 [38] to allow for comparison with the mental health measure drawn from electronic health records.

The Warwick Edinburgh Mental Wellbeing Scale (WEMWBS) is a 14-item scale with a scoring range of 1–5 and a total score ranging from 14–70. The recommended approach is to group the data using categorical approaches and to compare the scores with population norms. According to the scoring and analysis guidelines outlined in the WEMWBS website (https://warwick.ac.uk/fac/sci/med/research/platform/wemwbs/using), participant scores can be grouped as low (≤ 40), average (41–59) and high mental wellbeing (≥ 60). These cut-off points have been previously used by NHS direct, with a score of ≤ 40 indicating probable depression and ≥ 60 high mental wellbeing [39]. For the binary WEMWBS measure, the cut-off point was set at ≤ 40 to allow for a comparison between poor wellbeing and average to high wellbeing.

The presence of poor mental health was assessed through electronic healthcare records (EHR) using current treatment of anxiety or depression. Individuals who had received at least one prescription of an antidepressant, anxiolytic or hypnotic (according to the relevant British National Formulary (BNF) categories) during the one-year current period were considered grouped as being actively treated [38]. This measure was chosen over that of mental health diagnosis as we aimed to identify individuals who were receiving treatment at the time of the study, excluding those who might have had a historical diagnosis of anxiety or depression that would still be recorded in their EHR.

### Statistical analysis strategy

Multiple imputation using fully conditional specification (FCS), was implemented to deal with missing data, including all participants who had completed at least one of the three variables of interest (caring status, MHI-5 score, mental health treatment) (*N* = 1,069), resulting in the final cohort of 27,869 participants. IBM SPSS Statistics Version 25 was used to perform these imputations. We included all variables of interest in the imputation models; no additional variables linked to missingness were found in the available data. In total, 40 imputations (10 iterations in each instance) were created based on the missingness percentage of our dataset [40]. For continuous and scale variables, predictive mean matching was used, while for categorical or binary variables, logistic regressions were applied. For each variable, every other available variable was used to impute with no additional complex terms added. Multivariable logistic regression was used and results from imputed datasets were combined using Rubin’s rules [41].

**Fig. 1:**
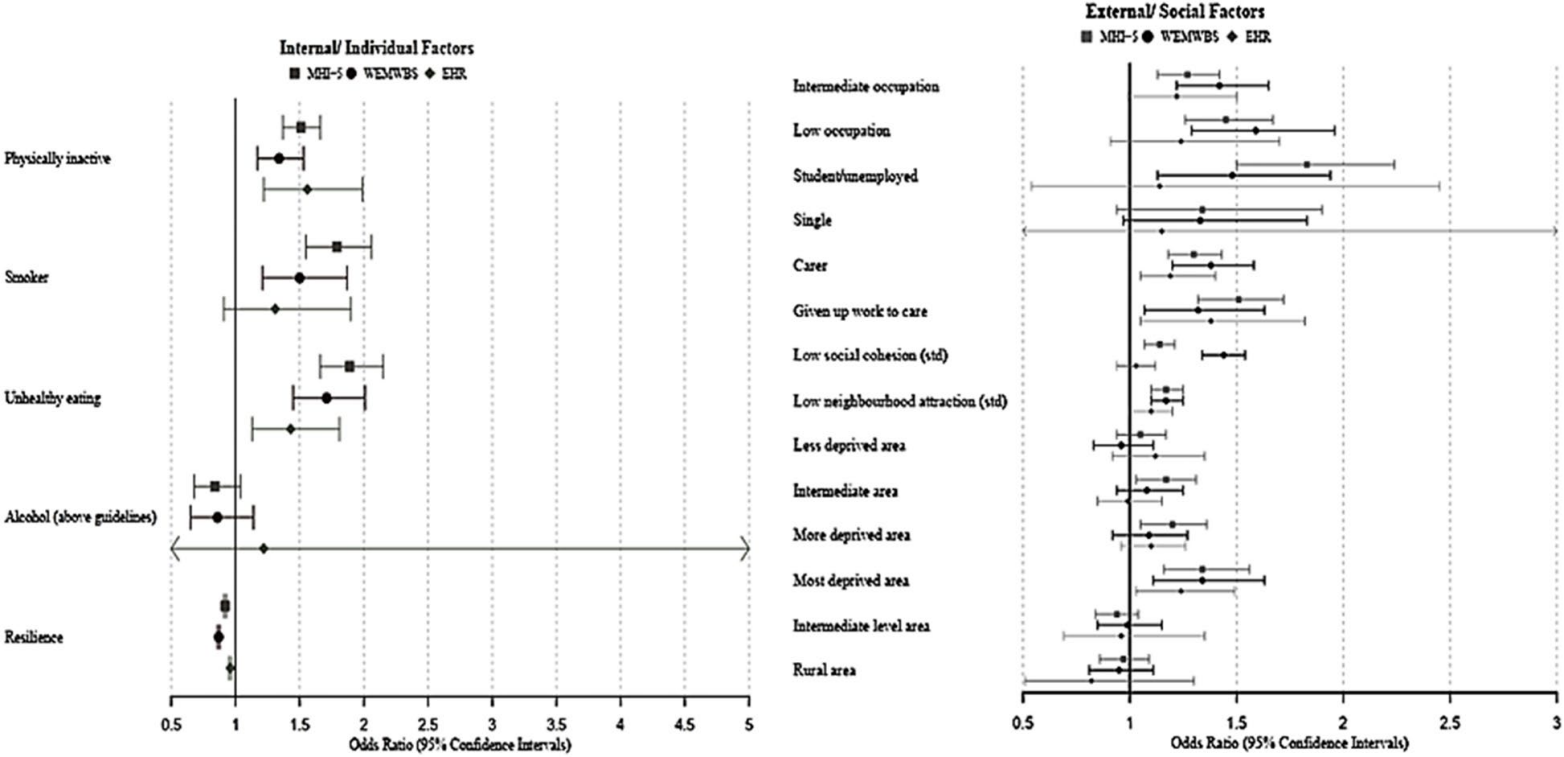
Individual and contextual factors in relation to MHI5, WEMWBS and EHR. Presenting Odds Ratios (95% Confidence Intervals) adjusting for gender, age and ethnicity. MHI-5: Five-question Mental Health Inventory-5; WEMWBS: Warwick Edinburgh Mental Wellbeing Scale; EHR: Electronic healthcare records

To explore how individual and contextual factors, including resilience and neighbourhood cohesion, were associated with mental health across the population, multivariate regressions, adjusting for age, gender and ethnicity, were performed, investigating the effect of individual and contextual factors (Fig. 1) in relation to mental health as assessed by the two self-report measures and mental health treatment.

The amount of variance explained by each set of factors was investigated by calculating Nested Nagelkerke *R*^2^ (equivalent of Nested *R*^2^ for binary outcomes) for each mental health measure (See Supplementary Table 3). This was done by calculating the mean of the Nagelkerke *R*^2^ for each imputed dataset which has been proposed as the appropriate method to use for multiple imputed datasets [42].

## Results

The final imputed dataset included 27,869 HWW participants with information in at least one of the key variables of interest. The imputed study cohort (see Table 1) included individuals across the age-range, with a higher proportion of middle-aged and older individuals (55–64 years: 18.9%). A greater proportion of our cohort was female (70.6%), of White background (98.2%) and with a high-level occupational status (higher occupations: 46.9%). The distribution of socio-demographic characteristics is consistent with other population surveys [28], reflecting the proportion of the population who are more likely to seek help from healthcare services. One in five (26%) were carers, of which most were providing only 1–9 h of care per week (18.3%). In addition, 10.2% of the cohort reported having to give up their work to care for someone. A large proportion of the cohort resided in urban settings (61.9%), while in terms of area deprivation our sample was broadly spread across all levels of deprivation, but with a trend towards fewer participants from most deprived areas (least deprived: 24%; intermediate: 21.4%; most deprived: 14.2%).

**Table 1:** Cohort characteristics. Presenting Numbers (Percentages) and Mean

Characteristics	Variable categories	Non-imputed data (Original cohort)	Imputed data (Summary statistics)
Age	16–24	2,281 (8.2)	2,281 (8.2)
	25–34	4,080 (14.6)	4,080 (14.6)
	35–44	4,403 (15.8)	4,403 (15.8)
	45–54	4,829 (17.4)	4,829 (17.4)
	55–64	5,275 (18.9)	5,275 (18.9)
	65–74	4,853 (17.4)	4,853 (17.4)
	75 +	2,148 (7.7)	2,148 (7.7)
Gender	Male	8,188 (29.4)	8,188 (29.4)
	Female	19,681 (70.6)	19,681 (70.6)
Ethnicity	White	17,294 (98.3)	27,379 (98.2)
	Non-White	296 (1.7)	490 (1.8)
Relationship status	In a relationship	10,326 (75.3)	19,644 (70.5)
	Single	3,388 (24.7)	8,225 (29.5)
Employment/ social class	Higher occupations	8,023 (51.5)	13,079 (46.9)
	Intermediate occupations	3,001 (19.3)	5,035 (18.1)
	Lower occupations	2,038 (13.1)	3,648 (13.1)
	Students or unemployed	2,523 (16.2)	6,107 (21.9)
Health behaviours	Drinking above guidelines	7,261 (44.2)	9,903 (35.5)
	Physically inactive	6,875 (42.7)	11,540 (41.4)
	Smoker	1,575 (9.6)	3,514 (12.6)
	Unhealthy eating	2,400 (15.4)	4,726 (17.0)
Caring status	Non-carer	11,821 (73.3)	20,618 (74.0)
	Carer 1–9 h	3,127 (19.4)	5,114 (18.3)
	Carer 20–49 h	502 (3.1)	860 (3.1)
	Carer ≥ 50 h	670 (4.2)	1,277 (4.6)
Given up work to care	No	14,322 (89.1)	25,027 (89.8)
	Yes	1,756 (10.9)	2,842 (10.2)
Area of settlement	Urban	16,850 (61.9)	17,248 (61.9)
	Intermediate	4,965 (18.2)	5,090 (18.3)
	Rural	5,406 (19.9)	5,531 (19.8)
Area deprivation	Least deprived	6,547 (24.1)	6,694 (24.0)
	Less deprived	6,168 (22.7)	6,312 (22.6)
	Intermediate	5,807 (21.3)	5,953 (21.4)
	More deprived	4,835 (17.8)	4,954 (17.8)
	Most deprived	3,864 (14.2)	3,956 (14.2)
MHI-5 score	No	11,088 (71.5)	20,135 (72.2)
	Yes	4,414 (28.5)	7,734 (27.8)
WEMWBS score	High/Good mental wellbeing	3,878 (78.5)	22,004 (79.0)
	Low mental wellbeing	1,064 (21.5)	5,865 (21.0)
Mental health treatment	No mental health treatment	22,502 (86.9)	23,693 (85.0)
	Mental health treatment	3,399 (13.1)	4,176 (15.0)
Resilience		72.4	72.8
Neighbourhood cohesion	Neighbourhood attraction	12.8	13.1
	Social cohesion	27.5	28.5

Although HWW participants were in general likely to report healthy lifestyles, 35.5% drank above alcohol guideline levels and 41.4% were physically inactive, while a smaller proportion were eating unhealthily and smoking (17.0% and 12.6% respectively). Over one in four self-reported poor mental health through the MHI-5 questionnaire (27.8%), while that proportion was lower for the WEMWBS scale (21%) and mental health treatment according to healthcare records (15%).

After adjustment, across all three mental health measures (Fig. 1), there was evidence of an association between both physical inactivity (MHI-5: adjusted Odds Ratio (OR_adj_) = 1.51 95%CI = 1.37–1.66; WEMWBS: OR_adj_ = 1.34 95%CI = 1.17–1.53; EHR: OR_adj_ = 1.56 95%CI = 1.22–1.99) and unhealthy eating (MHI-5: OR_adj_ = 1.89 95%CI = 1.66–2.15; WEMWBS: OR_adj_ = 1.71 95%CI = 1.45–2.01; EHR: OR_adj_ = 1.43 95%CI = 1.13–1.81) with poor mental health. However, there was only evidence (at a 5% significance level) of an association between smoking and poor mental health for the self-report mental health measures (MHI-5: OR_adj_ = 1.79 95%CI = 1.55–2.06; WEMWBS: OR_adj_ = 1.50 95%CI = 1.21–1.87) versus EHR (EHR: OR_adj_ = 1.31 95%CI = 0.91–1.90), while there was insufficient evidence at the 5% level of an association between drinking above guideline levels and poor mental health status.

In terms of resilience, higher levels of resilience were associated with better mental health across all measures, though the link was more prominent with self-report measures (MHI-5: OR_adj_ = 0.92 95%CI = 0.91–0.93; WEMWBS: OR_adj_ = 0.87 95%CI = 0.86–0.88; EHR: OR_adj_ = 0.96 95%CI = 0.95–0.97).

In these adjusted models, there was a reverse association between age and mental health status when comparing measures, with self-report being more likely among young people while receipt of treatment was more strongly linked with older age (See Supplementary Table 2). Furthermore, the association between female gender and poor mental health status was more prominent when using treatment as the dependent variable, compared to using the MHI-5. There was no statistically significant association between ethnicity and mental health status.

The associations between contextual factors and population mental health are shown in Fig. 1. Individuals with low level occupations were more likely to report poor mental health across self-report measures (MHI-5: OR_adj_ = 1.45 95%CI = 1.26–1.67; WEMWBS: OR_adj_ = 1.59 95%CI = 1.29–1.96). There was only evidence (at a 5% significance level) of an association between being a student or unemployed (MHI-5: OR_adj_ = 1.83 95%CI = 1.50–2.24; WEMWBS: OR_adj_ = 1.48 95%CI = 1.13–1.98) or a carer (MHI-5: OR_adj_ = 1.30 95%CI = 1.18–1.43; WEMWBS: OR_adj_ = 1.38 95%CI = 1.20–1.58) and poor self-reported mental health.

The potential effect of caring on employment status was further demonstrated with those giving up work to provide care being more likely to experience mental ill health across all three measures. Low social cohesion (MHI-5: OR_adj_ = 1.14 95%CI = 1.07–1.21; WEMWBS: OR_adj_ = 1.44 95%CI = 1.34–1.54) and low neighbourhood attraction (MHI-5: OR_adj_ = 1.17 95%CI = 1.10–1.25; WEMWBS: OR_adj_ = 1.17 95%CI = 1.10–1.25; EHR: OR_adj_ = 1.10 95%CI = 1.01–1.20) were also associated with poorer mental health, highlighting the close relationship between neighbourhood cohesion and mental health. However, when looking into other area level characteristics, there was evidence (at a 5% significance level) of an association between area level deprivation (Most deprived: MHI-5: OR_adj_ = 1.34 95%CI = 1.16–1.56; WEMWBS: OR_adj_ = 1.34 95%CI = 1.11–1.63; EHR: OR_adj_ = 1.24 95%CI = 1.03–1.49) and mental health as assessed by both self-report measures and EHR, though that was not the case for area of settlement.

**Fig. 2:**
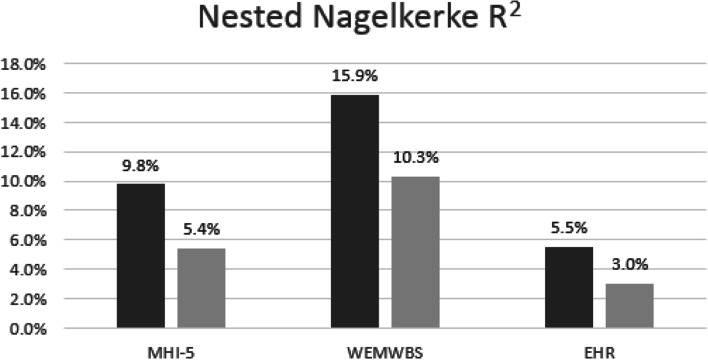
Variance explained by individual and contextual factors for each mental health measure. Presenting nested Nagerlkerke R2 (variance) explained by individual factors (in dark grey) and contextual factors (in light grey). MHI-5: Five-question Mental Health Inventory-5; WEMWBS: Warwick Edinburgh Mental Wellbeing Scale; EHR: Electronic healthcare records

Following the observation that individual and contextual factors differed in terms of their association with mental health across our population, it was further observed that across the three mental health measures, even after including caring (See Fig. 2) in the contextual factors, individual factors were able to explain almost double the amount of variance.

It has been reported that carers experience poorer physical and mental health due to challenges relating to their daily caring responsibilities. However, there is evidence that they also experience high satisfaction from caregiving, even in cases of high care demands, a disparity that has been attributed to intra- and interpersonal resources including resilience and social support [43]. Given that carers have been shown to differ from the general population, we further explored how these individual factors were linked to caring status by comparing carers with their non-carer peers using multivariate logistic regression analysis.

Table 2 shows that there was a gradient effect of age on caring status, with increasing age associated with increased likelihood of being a carer (35–44 years: OR_adj_ = 1.34 95%CI = 1.12–1.59; 75 + years: OR_adj_ = 2.36 95%CI = 1.79–2.98; Reference group: 16–24 years). Women were more likely to be carers (OR_adj_ = 1.40 95%CI = 1.27–1.54), while a reverse effect was observed for ethnicity, with those of Non-White background being less likely to report being carers (OR_adj_ = 0.70 95%CI = 0.50–0.97). Although carers have previously been shown to present with better health status [23], in the current study they were more likely to smoke (OR_adj_ = 1.27 95%CI = 1.09–1.47) and eat unhealthily (OR_adj_ = 1.16 95%CI = 1.03–1.29). Finally, there was insufficient evidence at the 5% level of an association between resilience and caring status (low resilience: OR_adj_ = 1.00 95%CI = 0.99–1.01).

**Table 2:** Individual factors among carers and non-carers. Presenting Numbers (Percentages) & Odds Ratios (95% Confidence Intervals)

	**Non-carers**	**Carers**	**Fully adjusted**
Age			
16–24	1,891 (9.2)	390 (5.4)	1.00
25–34	3,375 (16.4)	704 (9.8)	1.02 (0.86–1.21)
35–44	3,478 (16.8)	925 (12.7)	1.34 (1.12–1.59)
45–54	3,451 (16.7)	1,378 (19.0)	2.06 (1.70–2.49)
55–64	3,488 (16.9)	1,788 (24.6)	2.72 (2.23–3.32)
65–74	3,420 (16.6)	1,433 (19.8)	2.32 (1.89–2.84)
75 +	1,515 (7.4)	633 (8.7)	2.36 (1.87–2.98)
Gender			
Male	6,321 (30.7)	1,867 (25.7)	1.00
Female	14,297 (69.3)	5,384 (74.3)	1.40 (1.27–1.54)
Ethnicity			
White	20,217 (98.1)	7,161 (98.8)	1.00
Non-White	401 (1.9)	90 (1.2)	0.70 (0.50–0.97)
Health behaviours			
Drinking above guidelines	7,403 (35.9)	2,500 (34.5)	0.93 (0.77–1.12)
Physically inactive	8,373 (40.6)	3,167 (43.7)	1.02 (0.93–1.11)
Smoker	2,549 (12.4)	965 (13.3)	1.27 (1.09–1.47)
Unhealthy eating	3,509 (17.0)	1,217 (16.8)	1.16 (1.03–1.29)
Resilience	72.3 (mean)	72.5 (mean)	1.00 (0.99–1.01)

Following the observation that the effect of resilience within carers differs from that across the population, we replicated the analysis using the cluster of individual factors and the resilience sub-scales, which include individual, relational and contextual resilience resources (Table 3). There was evidence (at a 5% significance level) of an association between individual characteristics and mental health across all measures (MHI-5: OR_adj_ = 0.83 95%CI = 0.81–0.85; WEMWBS: OR_adj_ = 0.77 95%CI = 0.75–0.79; EHR: OR_adj_ = 0.92 95%CI = 0.89–0.95), indicating a close correlation between individual resilience and mental health. Furthermore, the measure exploring personal relationships was only associated with self-report measures (MHI-5: OR_adj_ = 0.95 95%CI = 0.92–0.99; WEMWBS: OR_adj_ = 0.92 95%CI = 0.90–0.95), while there was only evidence (at a 5% significance level) of an association between the contextual measures and mental health as assessed by WEMWBS (OR_adj_ = 0.93 95%CI = 0.91–0.96). It was also noted that the assessment of resilience as a binary construct vs a single continuous scale might be masking the amount of variance explained (Nested Nagelkerke *R*^2^) by individual factors across all mental health measures (e.g. MHI-5: binary *R*^2^: 3.6%; continuous *R*^2^: 7.3%; sub-scales derived *R*^2^: 9% and sub-constructs derived *R*^2^: 9.3%).

**Table 3:** Individual factors (using resilience sub-scales) in relation to MHI5, WEMWBS and treatment. Presenting Odds Ratios (95% Confidence Intervals) and Nested Nagelkerke *R*^2^

	**MHI-5**	**R**^**2**^	**WEMWBS**	**R**^**2**^	**EHR**	**R**^**2**^
**Binary resilience**	0.44 (0.39–0.48)	**0.036**	0.22 (0.19–0.25)	**0.098**	0.68 (0.60–0.77)	**0.007**
**Continuous scale**	0.92 (0.91–0.93)	**0.073**	0.87 (0.86–0.88)	**0.185**	0.96 (0.95–0.97)	**0.017**
**Sub-scales**		**0.090**		**0.201**		**0.021**
Individual	0.83 (0.81–0.85)		0.77 (0.75–0.79)		0.92 (0.89–0.95)	
Relational	0.95 (0.92–0.99)		0.92 (0.90–0.95)		0.99 (0.96–1.02)	
Contextual	1.00 (0.98–1.03)		0.93 (0.91–0.96)		0.99 (0.96–1.02)	
**Sub-constructs**		**0.093**		**0.210**		**0.031**
Individual Personal Skills	0.84 (0.79–0.89)		0.74 (0.70–0.78)		0.89 (0.84–0.94)	
Individual Peer Support	0.88 (0.83–0.94)		0.87 (0.81–0.92)		1.02 (0.94–1.11)	
Individual Social Skills	0.82 (0.77–0.87)		0.78 (0.73–0.83)		0.93 (0.85–1.02)	
Physical Caregiving	0.90 (0.79–1.03)		1.02 (0.90–1.15)		0.86 (0.75–0.98)	
Psychological Caregiving	0.96 (0.93–0.99)		0.90 (0.86–0.93)		1.02 (0.98–1.07)	
Context Spiritual	1.07 (1.03–1.11)		1.00 (0.96–1.05)		1.06 (1.01–1.12)	
Context Education	0.90 (0.83–0.97)		0.76 (0.72–0.81)		0.84 (0.77–0.92)	
Context Cultural	0.98 (0.94–1.03)		0.96 (0.91–1.01)		0.98 (0.92–1.04)	

## Discussion

This population-based study characterised putative contextual and individual risk factors associated with poor mental health and investigated their contribution to mental health status in a national population survey in Wales. Individual factors, including resilience and health behaviours, such as physical activity, were associated with the likelihood of experiencing poor mental health as assessed by both self-report measures and electronic healthcare records. Contextual factors, including neighbourhood cohesion and household and area level factors were also associated with poor mental health, though the associations were more prominent when using the self-report measures rather than mental health treatment. When both individual and contextual factors were included in the model, Individual factors were more closely associated (at a 5% significance level) with poor mental health and explained a greater amount of variance in relation to mental health across all measures, even after accounting for the role of caring responsibilities, which has been previously linked to mental ill health, as a contextual risk factor.

Across both individual and contextual factors, self-report measures captured population mental health better, showing a stronger association between each factor and mental health status. Resilience has been previously highlighted as a key factor closely linked to mental health across the population [6] and within carers [44] who tend to be more at risk of poor mental health when compared to their non-carer peers. In this context, resilience has been shown to relate to the way individuals respond to stress, adapt to arising problems and maintain their mental wellbeing [45]. When compared to contextual factors and particularly social cohesion and neighbourhood attraction which are two key aspects of neighbourhood cohesion [10], resilience showed a stronger association. This could suggest that neighbourhood conditions, such as deprivation and cohesion, might mediate the link between individual factors and poor mental health rather than increase the risk of mental ill health across the population, though a reverse association between mental health and neighbourhood perceptions has been proposed [46]. Interestingly, the strength of the association differed depending on the way the resilience construct was used. When multiple aspects of it were taken into consideration separately, resilience, especially individual resilience resources, was strongly linked to poor mental health suggesting that there is a complex interplay between a variety of personal skills, coping mechanisms and resilience. Social skills have been previously identified as moderators between stress and poor mental health during childhood [47], when personal resilience skills are developed shaping protective factors against mental ill health across the life span. This variation across sub-constructs is in line with previous studies proposing that resilience is a high-order concept with underlying components that might get swamped when it is used as a single-measure construct [48]. Relational and contextual factors can influence resilience (and vice versa), whilst resilience resources within individuals seem to be closely linked to the individual’s capacity to cope with stressful situations. This further highlights the need for a socioecological approach to resilience, which acknowledges the moderating role individual resilience can have between contextual factors and positive psychological outcomes [4]. In this cross-sectional study, it is also important to consider the potential bi-directional effect of this association. Whilst individuals with poor resilience resources might be more likely to experience poor mental health, individuals with poor mental health may experience maladaptive coping strategies and have reduced access to both intra- and interpersonal resources.

Unhealthy lifestyle behaviours have been linked to both poorer physical and mental health [49]. In the current study we observed that physical inactivity and unhealthy eating habits were strongly associated with poorer mental health, a finding that is in accordance with other population cohorts [50]. The role of smoking behaviours should not be neglected as there was evidence (at a 5% significance level) of an association with self-report measures of poor mental health and there was a trend towards a similar association in the EHR data. The observed variation between types of measures could relate to social desirability bias with respondents being reluctant to report unhealthy behaviours such as substance use in the context of a study focusing on population health and wellbeing [51]. This could also explain the difference in the direction of effect size observed in relation to unhealthy drinking, despite the lack of a significant association, with unhealthy drinking showing a protective link with mental health according to self-report measures but not mental health treatment.

In this study, the association between individual factors and mental ill health was more prominent than that of contextual factors, with both scales, MHI-5 and WEMWBS, capturing the association of interest more effectively than EHR. This finding could relate to the sensitivity and specificity of the three measures, as the two self-report questionnaires could be biased due to respondent or recall bias. These self-report scales have previously been used in community and healthcare settings to assess symptoms of depression and anxiety [52] and mental wellbeing [53]. Mental health treatment could capture more severe cases of mental ill health and could therefore exclude a proportion of the population who are experiencing poor mental health and have not yet taken the step to seek help from a healthcare professional or receive an appropriate diagnosis. The variation between measures could potentially be explained by the role of stigma in relation to help-seeking behaviours which has been identified across population sub-groups including ethnic minorities and individuals residing in rural areas [27].

The associations observed between demographic and lifestyle behaviours and individual and contextual psychosocial factors were also observed in the subset of carers. Caring status was associated with unhealthy behaviours, with carers being more likely to smoke and eat unhealthily, suggesting that they might not be looking after themselves due to factors related to their caring responsibilities [54]. This finding which contrasts with the suggestion that carers are intrinsically healthier than their non-carer peers, is supported by research demonstrating that only intense caring (providing care for more than 20 h per week), is linked to poor health status among carers [23] but could also be linked to the disproportionate number of older carers in our population which might bias the underlying age variation. In contrast, no significant variation was observed in terms of drinking above guidelines and physical inactivity, which could be attributed to either no differences between carers and non-carers in terms of drinking habits and physical activity or social desirability, resulting in a reluctance to report unhealthy behaviours that have been significantly discouraged through public health campaigns. Interestingly, the lack of association between caring and resilience is a finding that is contrary to what has been previously found in the relevant literature but could relate to the fact that the majority of studies on resilience of carers focus on carers of older adults [43], who tend to present with advanced illness, such as dementia, or be at the end of life [55]. Carers in our cohort might differ in terms of the type of health condition experienced by the person they care for, and it may be informative to collect further data to explore this.

### Strengths and limitations

This population-based study provides an overview of factors which are closely associated with mental health status of the population across Wales. The presence of mental ill health was assessed through the implementation of a robust record-linkage methodology that allowed for the comparison of both self-report questionnaires and healthcare records on treatment thus creating a more thorough assessment of population mental health. Similar levels of mental ill health were identified in our sample corresponding with variations that have been previously identified in relation to individual and area level characteristics, such as area deprivation [56]. Although the use of prescription data has been previously used as an indicator of mental ill health [57], it might miss individuals who might have had a past diagnosis of depression and anxiety or are in receipt of non-pharmacological treatment. Additional analyses (unpublished observations) using a case finding tool validated in a Welsh population [38] produced similar findings highlighting the usefulness of prescription data as a proxy for mental health status.

This study is limited, however, by the cross-sectional nature of the HWW questionnaires which do not allow for the discussion on directionality of observed associations with mental health. The collection of two-year follow-up measures which is currently underway would allow for the longitudinal analysis of the associations of interest and capture long term effects of individual and contextual factors more accurately. The skewed distribution of socio-demographic characteristics towards people who are female, of White background and higher occupations is consistent with other population surveys [28] but may limit the generalisability of the study findings. It is also important to note that no data on caring responsibilities such as the type of caring that was provided or the person towards whom it was directed was available. There is missing information on some key characteristics such as ethnicity or area deprivation, because no response is mandatory when participants complete a HWW questionnaire. However, this was accounted for as a multiple imputation methodology was implemented to address the issue of missingness and provide a more in-depth understanding of the association of interest in our sample. To prevent any skewness or inappropriate imputation of missing data, only participants for whom data were available on at least one of the three variables of interest, namely MHI-5 score, WEMWBS, mental health treatment and caring status, were included in the imputation model.

### Implications

This study highlights the presence of a stronger link between individual factors and mental health and wellbeing at the population level, when compared to contextual factors. Thus, targeting key aspects of resilience could help improve the quality of life of individuals who are experiencing chronic distress and poorer mental health, such as informal carers. Resilience-enabling interventions, which aim at the improvement of self-esteem and cognitions, can vary significantly and are quite flexible in their implementation as they can be used on a one-to-one basis or with groups of individuals, while they can also be adapted for online or telephone methods [4]. Interventions which provide skills-based learning relating to self-care may help mitigate the potential harm that unhealthy lifestyle behaviours can have on both physical and mental health. Supporting carers in this way could improve their daily life and enable them to undertake their day-to-day activities or provide better care to their care recipient while also caring for themselves. Additionally, promoting healthy eating behaviours could enhance current policy strategies targeting physical inactivity and other key lifestyle behaviours linked to wellbeing [58].

Moving from a deficit model of resilience towards a better understanding of the mechanisms and benefits of developing resilience skills and their positive reinforcement could help promote environments which enable individuals to cope with stressful situations. This approach may be particularly important for young people whose mental health is influenced by personal, social and resilience resources [47]. Improving knowledge and skills around these factors may better equip people to cope with contextual or relational risk factors, including cultural variations and physical caregiving which can act cumulatively to increase the risk of poor mental health. Policy frameworks which reduce the risk of adverse events or provide support to deal with them may be helpful in developing positive coping behaviours, resilience and reducing mental ill health. Steps to promote psychological resilience should be taken within the context of a socio-ecological understanding of resilience, accounting for a range of contextual and cultural factors, such as the individual’s social environment. These can change over the life course, highlighting the need for an approach that utilises individual and systemic resources towards achieving lifelong psychological resilience and wellbeing.

## Conclusion

This population-based study found that individual factors are more intrinsically linked to mental health than contextual factors. It also highlighted resilience, when examined as a complex set of skills and resources, to be more closely associated with mental ill health than neighbourhood or social cohesion. These findings contribute to greater understanding of the interplay between individual and contextual factors by examining their independent effects along with their potentially cumulative effect on mental health. Future research and clinical practice could benefit from considering the complexities of resilience as a socio-ecological construct, especially when focusing on population subgroups who are more at risk of experiencing poor mental health (such as carers), as well as accounting for the potential role of existing underlying mechanisms which interact with extrinsic factors (such as neighbourhood environment) leading to different pathways of mental ill health across the population.

## Supplementary Information

**Additional file 1.**

## Data Availability

The datasets generated and/or analysed during the current study are not publicly available due to privacy and/or ethical restrictions, but data that support the findings of this study are available from the corresponding author on reasonable request.

## Abbreviations

HWW: HealthWise Wales
SAIL: Secure Anonymised Information Linkage
REC: Research Ethics Committee
SAPPHIRe: Secure Analysis Portal and Protected HWW Information Repository
CMD: Common mental health disorders
GPPAQ: General Practice Physical Activity Questionnaire
RRC-ARM: Resilience Research Centre Adult Resilience Measure
NS-SEC: National Statistics Socio-Economic Classification
MHI-5: Five-question Mental Health Inventory
WEMWBS: Warwick Edinburgh Mental Wellbeing Scale
EHR: Electronic healthcare records
BNF: British National Formulary
FCS: Fully conditional specification
OR_adj_: Adjusted Odds Ratio
95%CI: 95% Confidence interval
