# Supplementary material for: The association between resilience resources, contextual factors and mental health status: a national population-based study
Source: BMC Public Health. 2022 Mar 29;22:602. doi: 10.1186/s12889-022-13013-2 (PMC8962564; doi:10.1186/s12889-022-13013-2)
Supplement: Supplementary file 1 — Additional file 1. [file 12889_2022_13013_MOESM1_ESM.docx]

**Supplemental Material**

**
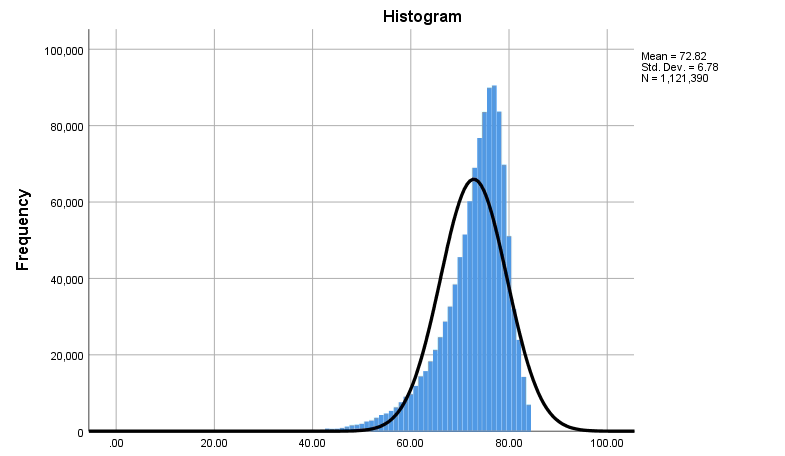
**

**Supplementary Figure 1** Distribution of resilience score across the study population

**Constructing neighbourhood cohesion scale**

In the unimputed dataset, we analysed data from individuals who have answered all 17 stems of the Neighbourhood Cohesion scale. Following a single-level principal component analysis, we produced a scree plot (Supplementary Figure 2) and looked for an “elbow” in this picture.


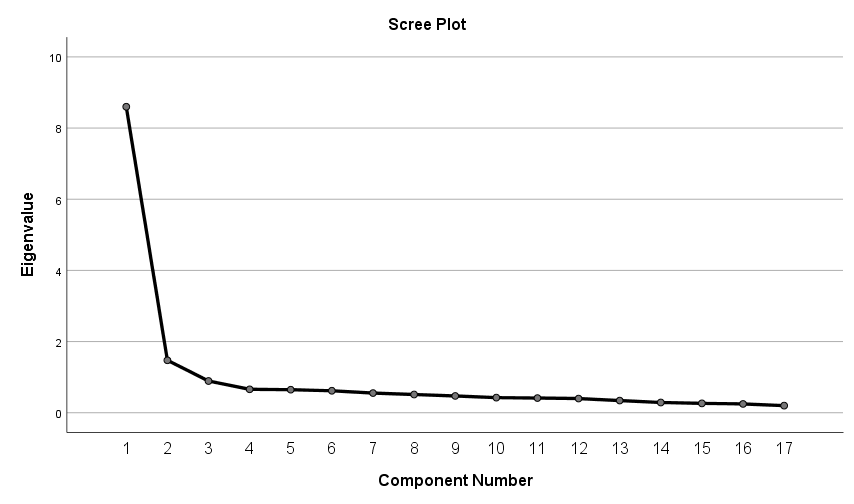


**Supplementary Figure 2** Screen plot from Principal Component Analysis. The eigenvalues constitute the proportion of variance explained by each component, plotted against component number and order by decreasing eigenvalue.

We determined that a two-factor solution was an appropriate simplification of the adapted neighbourhood cohesion questionnaire items. These factors accounted respectively for 32% and 26% of the item-level variability; the factor loadings are shown in Supplementary Table 1.

**Supplementary Table 1** Factor loading for the two-factor solution, following a varimax rotation (Spearman’s rank)

| Item | Factor 1 | Factor 2 | Subscale | Item-subscale correlation |
| --- | --- | --- | --- | --- |
| 1 | 0.249 | 0.833 | NB | 0.820 |
| 2 | 0.484 | 0.685 | NB | 0.812 |
| 3 | 0.627 | 0.152 | SC | 0.638 |
| 4 | 0.713 | 0.359 | SC | 0.786 |
| 5 | 0.179 | 0.835 | NB | 0.826 |
| 6* | 0.676 | 0.312 | SC | 0.721 |
| 7 | 0.761 | 0.259 | SC | 0.785 |
| 8 | 0.594 | 0.357 | SC | 0.669 |
| 9* | 0.720 | 0.423 | SC | 0.810 |
| 10 | 0.751 | 0.177 | SC | 0.748 |
| 11 | 0.531 | 0.283 | SC | 0.591 |
| 12 | 0.229 | 0.815 | NB | 0.830 |
| 13 | 0.436 | 0.591 | NB | 0.730 |
| 14 | 0.688 | 0.119 | SC | 0.700 |
| 15 | 0.596 | 0.319 | SC | 0.689 |
| 16 | 0.663 | 0.543 | SC* | 0.819 |
| 17 | 0.285 | 0.692 | NB | 0.727 |

We partitioned the items into the two factors according to the greater factor loading on individual items. In this instance, if a factor loading on a particular component exceeded 0.5, we included a question item into that component. 11 questions were included in the first component; the two largest factor loading were for items 7 (If I need advice about something – can go to someone in my neighbourhood) and 10 (I borrow things and exchange favours with my neighbours). As these related to the “degree of interaction within the neighbourhood” construct proposed by Buckner, the component was labelled “social cohesion”. 7 items were identified in the second component; the two largest factor loadings were for the items 1 (I am attracted to living in this neighbourhood) and 5 (Given the opportunity, I would like to move out of this neighbourhood, [reverse coded]). As these related to the “degree of attraction to the neighbourhood” originally proposed by Buckner, this component was labelled “neighbourhood attraction”.

**Supplementary Table 2** Presenting numbers from Figure 1

|  | **MHI-5** | **WEMWBS** | **EHR** |
| --- | --- | --- | --- |
|  | Individual | | |
| Age |  |  |  |
| 25-34 | 0.70 (0.60-0.81) | 0.87 (0.72-1.06) | 1.60 (1.26-2.02) |
| 35-44 | 0.58 (0.50-0.67) | 0.79 (0.65-0.95) | 2.01 (1.53-2.65) |
| 45-54 | 0.51 (0.44-0.60) | 0.73 (0.60-0.89) | 2.79 (2.17-3.58) |
| 55-64 | 0.41 (0.34-0.49) | 0.58 (0.47-0.71) | 2.87 (2.29-3.60) |
| 65-74 | 0.26 (0.21-0.31) | 0.38 (0.31-0.48) | 2.48 (1.70-3.63) |
| 75+ | 0.22 (0.17-0.28) | 0.32 (0.30-0.43) | 2.54 (1.39-4.63) |
| Gender |  |  |  |
| Female | 1.31 (1.17-1.45) | 1.23 (1.09-1.40) | 1.58 (1.32-1.90) |
| Ethnicity |  |  |  |
| Non-White | 1.27 (0.93-1.75) | 0.87 (0.59-1.29) | 0.88 (0.63-1.23) |
| Unhealthy behaviours |  |  |  |
| Physically inactive | 1.51 (1.37-1.66) | 1.34 (1.17-1.53) | 1.56 (1.22-1.99) |
| Smoker | 1.79 (1.55-2.06) | 1.50 (1.21-1.87) | 1.31 (0.91-1.90) |
| Unhealthy eating | 1.89 (1.66-2.15) | 1.71 (1.45-2.01) | 1.43 (1.13-1.81) |
| Alcohol (above guidelines) | 0.84 (0.68-1.04) | 0.86 (0.65-1.14) | 1.22 (0.27-5.44) |
| Resilience |  |  |  |
| High resilience | 0.92(0.91-0.93) | 0.87 (0.86-0.88) | 0.96 (0.95-0.97) |
|  | Contextual | | |
| Age |  |  |  |
| 25-34 | 0.75 (0.65-0.86) | 0.91 (0.76-1.09) | 1.62 (1.26-2.07) |
| 35-44 | 0.63 (0.53-0.74) | 0.86 (0.71-1.04) | 2.05 (1.54-2.72) |
| 45-54 | 0.54 (0.46-0.64) | 0.79 (0.64-0.96) | 2.83 (2.16-3.72) |
| 55-64 | 0.41 (0.37-0.51) | 0.64 (0.52-0.78) | 2.88 (2.28-3.65) |
| 65-74 | 0.24 (0.19-0.30) | 0.42 (0.33-0.54) | 2.45 (1.69-3.55) |
| 75+ | 0.20 (0.15-0.26) | 0.34 (0.25-0.46) | 2.44 (1.37-4.35) |
| Gender |  |  |  |
| Female | 1.17 (1.05-1.31) | 1.07 (0.95-1.21) | 1.51 (1.29-1.76) |
| Ethnicity |  |  |  |
| Non-White | 1.26 (0.95-1.69) | 0.94 (0.65-1.38) | 0.86 (0.63-1.19) |
| Occupation (high) |  |  |  |
| Intermediate occupation | 1.27 (1.13-1.42) | 1.42 (1.22-1.65) | 1.22 (0.99-1.50) |
| Low occupation | 1.45 (1.26-1.67) | 1.59 (1.29-1.96) | 1.24 (0.91-1.70) |
| Student/Unemployed | 1.83 (1.50-2.24) | 1.48 (1.13-1.94) | 1.14 (0.54-2.45) |
| Household |  |  |  |
| Single | 1.34 (0.94-1.90) | 1.33 (0.97-1.83) | 1.15 (0.43-3.06) |
| Carer | 1.30 (1.18-1.43) | 1.38 (1.20-1.58) | 1.19 (1.05-1.40) |
| Given up work to care | 1.51 (1.32-1.72) | 1.32 (1.07-1.63) | 1.38 (1.05-1.82) |
| Neighbourhood cohesion |  |  |  |
| Low cohesion (std) | 1.14 (1.07-1.21) | 1.44 (1.34-1.54) | 1.03 (0.94-1.12) |
| Poor attraction (std) | 1.17 (1.10-1.25) | 1.17 (1.10-1.25) | 1.10 (1.01-1.20) |
| Area deprivation (least) |  |  |  |
| Less deprived | 1.05 (0.94-1.17) | 0.96 (0.83-1.11) | 1.12 (0.92-1.35) |
| Intermediate | 1.17 (1.03-1.31) | 1.08 (0.94-1.25) | 0.99 (0.85-1.15) |
| More deprived | 1.20 (1.05-1.36) | 1.09 (0.92-1.27) | 1.10 (0.96-1.26) |
| Most deprived | 1.34 (1.16-1.56) | 1.34 (1.11-1.63) | 1.24 (1.03-1.49) |
| Settlement (urban) |  |  |  |
| Intermediate level area | 0.94 (0.84-1.04) | 0.99 (0.85-1.15) | 0.96 (0.69-1.35) |
| Rural area | 0.97 (0.86-1.09) | 0.95 (0.81-1.11) | 0.82 (0.51-1.30) |

**Supplementary Table 3** Presenting full and nested regression model estimates from Figure 2

INDIVIDUAL MHI5

Full model Nagerlkerke R2: 0.218/ no individual factors Nagerlkerke R2: 0.120 / Nested Nagerlkerke R2: 0.098 = individual factors account for 9.8% of variance

*INDIVIDUAL WEMWBS*

Full model Nagerlkerke R2: 0.278/ no individual factors Nagerlkerke R2: 0.119 / Nested Nagerlkerke R2: 0.159= individual factors account for 15.9% of variance

INDIVIDUAL EHR

Full model Nagerlkerke R2: 0.111 / no individual factors Nagerlkerke R2: 0.056 / Nested Nagerlkerke R2: 0.055= individual factors account for 5.5% of variance

CONTEXTUAL MHI5

Full model Nagerlkerke R2: 0.218 / no contextual factors Nagerlkerke R2: 0.164 / Nested Nagerlkerke R2: 0.054= contextual factors account for 5.4% of variance

CONTEXTUAL WEMWBS

Full model Nagerlkerke R2: 0.278 / no contextual factors Nagerlkerke R2: 0.175 / Nested Nagerlkerke R2: 0.103 = contextual factors account for 10.3% of variance

CONTEXTUAL EHR

Full model Nagerlkerke R2: 0.111 / no contextual factors Nagerlkerke R2: 0.081 / Nested Nagerlkerke R2: 0.030 = contextual factors account for 3.0% of variance
